# Integrating Multi-Domain Approach for Identification of Neo Anti-DHPS Inhibitors Against Pathogenic *Stenotrophomonas maltophilia*

**DOI:** 10.3390/biology14081030

**Published:** 2025-08-11

**Authors:** Alhumaidi Alabbas

**Affiliations:** Department of Pharmaceutical Chemistry, College of Pharmacy, Prince Sattam Bin Abdulaziz University, Al-Kharj 16273, Saudi Arabia; ab.alabbas@psau.edu.sa; Tel.: +966-50-881-6604 or +966-11-588-8888 (ext. 6013)

**Keywords:** DHPS, entropy energy, salt bridge, virtual screening, post-simulation analysis

## Abstract

Background: The increasing number of resistant bacterial strains is reducing the effectiveness of antimicrobial drugs in preventing infections. It has been shown that resistant strains invade living organisms and cause a wide range of illnesses, leading to a surprisingly high death rate. Objective: The present study aimed to identify novel dihydropteroate synthase (DHPS) inhibitors from *Stenotrophomonas maltophilia* using structure-based computational techniques. Methodology: This in silico study used various bioinformatics and cheminformatics approaches to find new DHPS inhibitors. It began by retrieving the crystal structure via PDB ID: 7L6P, followed by energy minimization. The DHPS enzyme was virtually screened against the CHEMBL library to target *S. maltophilia* through enzyme inhibition. Then, absorption, distribution, metabolism, and excretion (ADME) analysis was performed to select the top hits. This process identified the top-10 hits. Additionally, imidazole (control) was used for comparative assessment. Furthermore, a 100 ns molecular dynamics simulation and post-simulation analyses were conducted. The docking results were validated through binding free energy calculations and entropy energy estimation approaches. Results: The docking results prioritized 10 compounds based on their binding scores, with a maximum threshold of −7 kcal/mol for selection. The ADME assessment shortlisted 3 out of 10 compounds: CHEMBL2322256, CHEMBL2316475, and CHEMBL2334441. These compounds satisfied Lipinski’s rule of five and were considered drug-like. The identified inhibitors demonstrated greater stability and less deviation compared to the control (imidazole). The average RMSD stayed below 2 Å, indicating overall stability without major deviations in the DHPS–ligand complexes. Post-simulation analysis assessed the stability and interaction profiles of the complexes under physiological conditions. Hydrogen bonding analysis showed the control to be more stable than the three tested complexes. Increased salt bridge interactions suggested stronger electrostatic stabilization, while less alteration of the protein’s secondary structure indicated better structural compatibility. These findings support the potential of these novel ligands as potent DHPS inhibitors. Binding energy estimates showed that CHEMBL2322256 was the most stable, with scores of −126.49 and −124.49 kcal/mol. Entropy calculations corroborated these results, indicating that CHEMBL2322256 had an estimated entropy of 8.63 kcal/mol. Conclusions: The newly identified compounds showed more promising results compared to the control. While these compounds have potential as innovative drugs, further research is needed to confirm their effectiveness as anti-DHPS agents against antibiotic resistance and *S. maltophilia* infections.

## Simple Summary

The increasing antibiotic resistance of bacteria reduces the effectiveness of antimicrobial drugs in preventing infections. This study reports novel dihydropteroate synthase (DHPS) inhibitors using computational techniques. CHEMBL2322256, CHEMBL2316475, and CHEMBL2334441 were considered promising drug-like molecules against the target protein. The identified inhibitors demonstrated greater stability and less deviation compared to the control (imidazole). Binding energy estimates showed that CHEMBL2322256 formed the most stable complex with the enzyme. Entropy calculations corroborated these results. The newly identified compounds showed more promising results compared to the control.

## 1. Introduction

*Stenotrophomonas maltophilia (S. maltophilia*) is a Gram-negative bacillus and a non-fermenting bacterium [1]. It can be found in a variety of environmental sources, such as soil, plants, and animals. It is also prevalent in aquatic environments [2]. Since *S. maltophilia* can form a biofilm on surfaces like catheters, mechanical ventilators, and feeding tubes, it is known to be the sole species of the *Stenotrophomonas* genus that can infect humans [3]. These infections primarily affect hospitalized and immunocompromised patients, and especially intensive care unit (ICU) patients who have had prosthetic devices installed [3]. The bacterium has been reported to be the most common organism isolated in clinical laboratories, after *Acinetobacter* spp., *Pseudomonas aeruginosa*, and the *Burkholderia cepacia* complex [4]. In addition to blood infections like bacteremia, *S. maltophilia* can also cause wound and soft tissue infections, peritonitis, meningitis, urinary tract infections (UTIs), as well as bone/joint infections [5]. The majority of infections caused by the aforementioned bacterium occur in the lower respiratory tract as tracheobronchitis or pneumonia due to mechanical ventilation [6]. The infections have a very high death rate; when linked to pneumonia, the rate is 75%, and when linked to bacteremia, it is 20% [6]. Using a variety of methods, such as the synthesis of enzymes that immobilize erythromycin and aminoglycoside acetyltransferase, the bacterium is resistant to various antimicrobials. Furthermore, several genes that generate efflux pumps may be present in *S. maltophilia*. Resistance may arise naturally or be acquired by mutations or horizontal gene transfer of the resistant genes [7].

Additionally, pan-resistant strains of *S. maltophilia* have been identified in hospitals due to the improper administration of antibiotics, particularly broad-spectrum antibiotics [8]. Additionally, the bacterium exhibits multidrug resistance (MDR) to specific antibiotics [8]. There are not many choices for treating its infections since it is resistant to numerous ß-lactam antibiotics, including carbapenem or aminoglycosides [9]. The occurrence of multidrug-resistant (MDR) bacteria, including *S. maltophilia*, has increased in tandem with the severe acute respiratory syndrome coronavirus 2 (CoV2) epidemic that began in Wuhan at the end of 2019 [10]. Because of its innate prolonged antibiotic resistance, it has limited treatment choices, and the only major antibiotic that is advised is sulfamethoxazole/trimethoprim (SXT). Yet, the global rise of SXT resistance has been linked to the transmission of dihydropteroate synthase (DHPS) genes, such as *sul1* and *sul2*; hence, it is critically important to develop a rapid, sensitive, and cost-effective method for detecting the distribution of *sul* genes [11]. This microorganism’s decreased reactivity to antibiotics is mostly due to the presence of genes in its chromosome that encode efflux pumps and enzymes that neutralize drugs [12]. Therefore, there are not numerous options to treat these illnesses. Antimicrobial drugs are drawn to DHPS since it is an essential enzyme in the folate biosynthesis pathway that is not present in humans but is essential for bacterial survival [13]. A high failure rate, prolonged development times, and exorbitant costs are some of the main problems with traditional drug design. These issues have been caused by the need for lengthy screening processes, a dearth of therapeutic options, and challenges in accurately predicting a person’s drug safety and efficacy [14]. The goal of the time-consuming and expensive drug discovery process is to create novel medication candidates [15]. The most common method of accomplishing this has been to utilize computational techniques through the preclinical phase of drug research. The term computer-aided drug design (CADD) refers to computational techniques for identifying, developing, and assessing medications and active ingredients with similar biological properties [16]. Molecular docking, molecular dynamics simulation and pharmacokinetics properties prediction are the main components of CADD [17]. The current study is focused on combating antibiotic resistance triggered by bacteria with the dihydropteroate synthase. This study utilized different cheminformatics and bioinformatics approaches for the identification of novel inhibitory drug candidates.

## 2. Methodology

**Figure 1:**
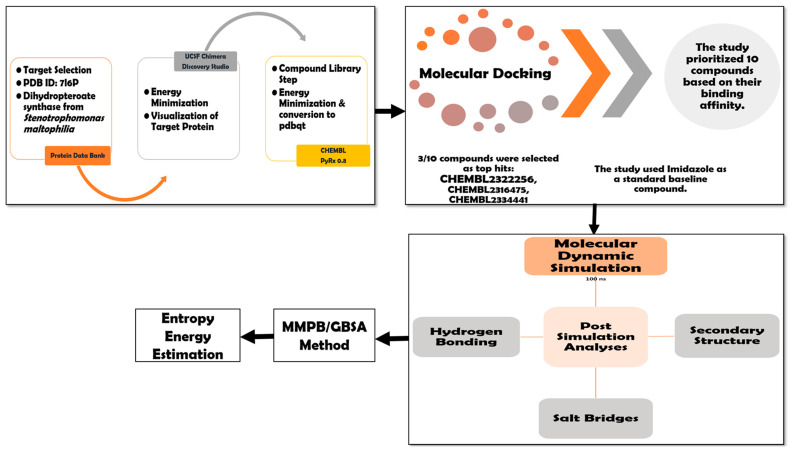
Depicts the hierarchical order of the methodology adopted in the current study. The workflow includes (i) target selection, (ii) energy minimization, and (iii) library preparation, proceeding to (iv) in silico molecular docking and (v) molecular dynamic solation. Then comes post-simulation analysis, including (vi) hydrogen bonding analysis, (vii) salt bridges studies, and (viii) secondary structure analysis. Afterwards, the validation of the docking results was performed by (ix) MMPB/GBSA methods and (x) entropy energy estimation.

The method flow used in the study is given in Figure 1.

### 2.1. Target Identification and Energy Minimization Phase

The crystal structure of the receptor molecules *S. maltophilia* dihydropteroate synthase (DHPS) via PDB ID: 7l6P was retrieved from the Protein Data Bank (PDB) https://www.rcsb.org/ (accessed on 12 January 2025). The protein preparation was carried out using the standard procedure, which involved eliminating the co-crystallized ligand, adding cofactors and polar hydrogens, and removing water molecules. This step is energy minimization performed through UCSF Chimera. Furthermore, the Discovery Studio v2021 was used to visualize the receptor molecule [18].

### 2.2. Compound Library Selection and Preparation Phase

A compound library named Chemical Entities of Biological and Medicinal Interest (CHEMBL), consisting of 2496 compounds, was used against the target receptor DHPS to identify novel inhibitory compounds. The library was saved in a structure data file (SDF) format [19]. Afterwards, it was imported into the docking software PyRx 0.8. All the compounds were energy-minimized and then converted into pdbqt format [20]. The current study utilized imidazole as a control for comparative assessment during the in silico investigation (molecular docking and MD simulation). Imidazole’s previously documented binding to the DHPS of *S. maltophilia*, the same bacterial species being studied, led to its selection as the study’s control compound. Its function as a site-bound ligand was confirmed by the crystal structure of DHPS complexed with imidazole (PDB ID: 7L6P), which provides a solid structural foundation for its use as a positive control in our computational research. Crystal structure of DHPS with active-site-bound imidazole PDB ID: 7L6P.

### 2.3. Molecular Docking Step

Molecular docking is a technique used in computer-aided drug design (CADD) that determines binding affinities by using scoring functions to determine how a tiny molecule/compound (ligand) interacts with the target protein/receptor within its active pockets [21]. The compound library containing 2496 compounds underwent an extensive virtual screening procedure via the PyRx 0.8 Auto Dock Vina tool. The compounds were ranked and selected as top hits based on the binding affinity score [22]. Moreover, the classification of the compounds revealed key interactions such as hydrogen bonding and hydrophobic and hydrophilic contacts [23]. The top hits’ chemical structure and chemical name were drawn through ChemDraw Ultra 12.0 software [24]. The visualization (3D) was carried out with PyMol v3.1 software and Discovery Studio v2021 [25].

### 2.4. ADME Property Prediction Step

The top-ranked compounds were assessed for evaluation of their absorption, distribution, metabolism, and excretion in an ADME profile [26]. The top-ranked compounds’ pharmacokinetics, lipophilicity, drug-likeness, and Lipinski’s rule of 5 criteria, as well as their pharmacokinetic properties, were predicted using the SwissADME. http://www.swissadme.ch/ (accessed on 28 January 2025) [27].

### 2.5. Molecular Dynamics (MD) Simulation

MD simulations demonstrated the time-dependent movement of molecules in the DHPS–ligand complex under physiological conditions [28]. The MD simulation was performed for 100 ns through the AMBER package v21 in three steps. The primary step included the preparation step, in which DHPS–ligand complexes were prepared; subsequently, there was the pre-processing phase, and lastly, the production run [29]. The MD trajectories were visualized through the xmgrace tool of AMBER [30].

### 2.6. Hydrogen Bonding Analysis

The hydrogen bonds generated during the MD simulation were estimated using post-simulation analysis, known as hydrogen bonding studies from the MD trajectories. The analysis evaluated the number of frames, occupancy rate, number of donors, number of acceptors, bond angle and average distance between the residues and the ligands [31]. These interactions enhance the protein–ligand complex’s stability and specificity. The hydrogen bonds were analyzed from the MD trajectories.

### 2.7. Salt Bridges Analysis

Salt bridges are electrostatic interactions formed between oppositely charged residues and stabilize the protein structure [32]. The salt bridges analysis was performed from the MD trajectories by using the vmd module of the Amber package [33].

### 2.8. Secondary Structure Analysis

The alterations in the alpha-helix, beta-sheets, coils, and twists of the protein structure were identified via secondary structure studies of the PL complexes. The aforementioned research provided a better understanding of the dynamic behavior of protein–ligand complexes in CADD. This investigation evaluated how the protein structure changes after a ligand binds to its alpha-helix, beta-sheets, twists, and coils [34]. The analysis was carried out using sec.in and the Python script of Amber software [35].

### 2.9. MMPB/GBSA Calculations

MMPB/GBSA analysis was employed to determine the binding free energy of the DHPS–compound complexes all over the interaction [36]. Molecular mechanics Poisson–Boltzmann/generalized Born surface area (MMPB/GBSA) was the method employed for calculating the binding free energy during the MD simulation [37].

The following equation was used in the calculations:

∆Gbinding = ∆Eele + ∆Evdw + ∆Gpol + ∆Gnp

ΔGbinding is the binding free energy of complexes, whilst ΔEele, ΔGpol, ΔGnp, and ΔEvdw represent the fluctuating electrostatic energy, non-polar solvation energy, polar solvation energy, and van der Waals energy, correspondingly, in the equation above. To calculate different energy transition factors, the solvent along with solute dielectric constants was utilized.

### 2.10. Entropy Energy Calculations

One crucial stage of the in silico drug design method is entropy energy estimation. Entropy measures the instability and randomness of a system [38]. Entropy describes how the particles and energy are arranged in a system [39]. The PL complexes and their binding interaction mode were described by this method. For calculating the entropy energy of each compound, the AMBER program was utilized.

## 3. Results

### 3.1. Target Receptor Identification and Preparation Phase

**Figure 2:**
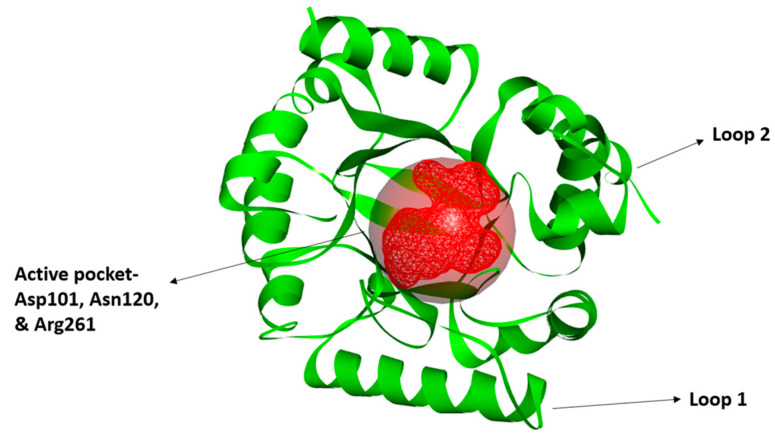
The crystal structure of DHPS, showing active pockets involved in molecular docking studies. The protein is shown in the green cartoon illustration, while the binding pockets are in the red surface, including the main active site residues (Asp101, Asn120, and Arg261). The Loop 1 and Loop 2 are also represented, which are structurally vital.

The crystal structure of the enzyme was obtained through PDB ID: 7l6p. The structure was determined using the X-ray diffraction technique with a resolution of 2.35 Å. Furthermore, the structure shows Global Symmetry: Cyclic-C2, Global Stoichiometry: Homo 2-mer-A2. The energy minimization step was performed, which removed steric clashes, and a stable protein structure was achieved. The protein was saved in PDB format subsequently. The active site residues were Asp101, Asn120, and Arg261, obtained via the literature review. The three-dimensional (3D) structure, labelled with the active sites, is shown in Figure 2.

### 3.2. Molecular Docking Findings and Binding Affinity Score of Compounds Against DHPS

A compound library containing 2496 compounds was efficiently screened against the DHPS. Compounds with the highest binding affinities and smallest docking scores had their docking conformations visualized [40]. The docking threshold was set to −7 kcal/mol. The top-ten compounds were shortlisted based on their binding affinity scores. The top hits, along with necessary information, are shown in Table 1.

### 3.3. Molecular Interaction Between DHPS and Top-3 Hits

**Figure 3:**
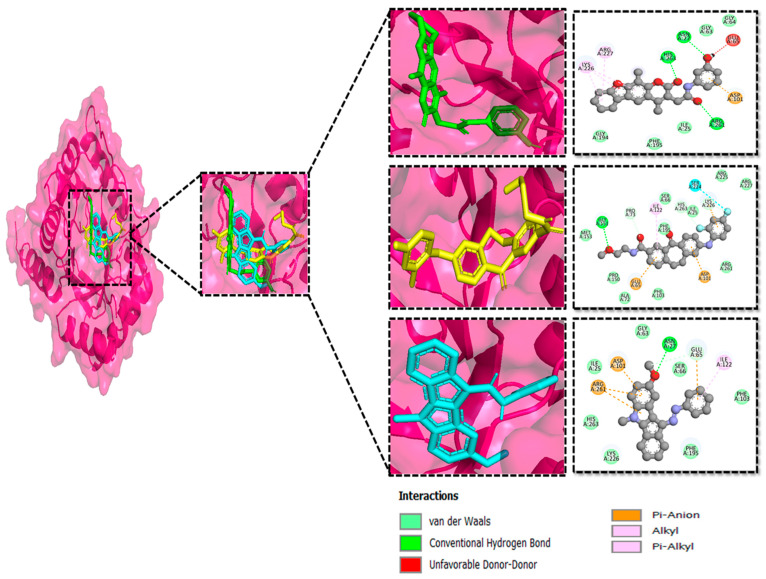
The DHPS–ligand interaction profile of the top-three hits. The top-1-CHEMBL2322256 (green), top-2-CHEMBL2316475 (yellow), and top-3-CHEMBL2334441 (cyan). For the 2D interaction maps, the dark green is conventional hydrogen bonds, light green depicts van der Waals bonds, red is unfavorable bumps, orange represents pi–anion, light pink shows alkyl and pi–alkyl, and cyan shows fluorine.

The protein–ligand interaction patterns (2D and 3D) for DHPS and the three possible candidates are shown in Figure 3. The first-ranked compound is CHEMBL2322256, based on the most favorable binding affinity (−8.3 kcal/mol), which interacted with Asn27, His263, and Arg61 and created hydrogen bonds. Moreover, the protein formed van der Waals interactions with GLY194, PHE195, ILE25, and GLY64. The second selected compound, CHEMBL2316475 (−7.8 kcal/mol), interacted with Gly151 via hydrogen bonds and created van der Waals bonds with Met153, Pro150, Ala72, Arg261, Pro73, Ser66, His263, Ile25, Lys226, Arg227, and Arg261. The third ranked compound, named CHEMBL2334441, with the binding affinity of −7.6 kcal/mol, exhibited hydrogen bonds with Asn27, while van der Waals interaction was seen with Ile25, Gly63, Glu65, His263, Phe103, Phe195, Lys226, and Ser66.

### 3.4. Molecular Docking Analysis of the Control as a Baseline Compound

**Figure 4:**
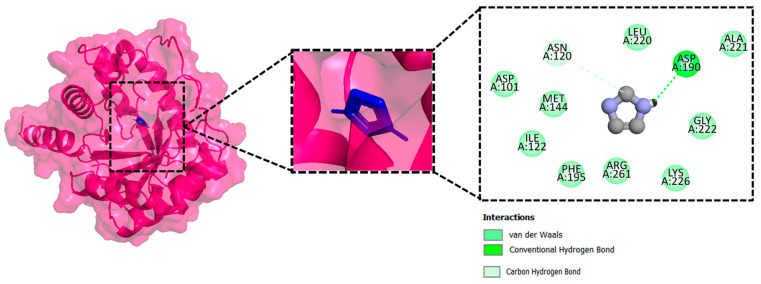
The 3D interaction (**left side**) and 2D interaction (**right side**) of DHPS with the imidazole (control).

To evaluate the potential of the novel identified compounds, a reference compound was used as a standard drug against DHPS. The reference compound, named imidazole (2,3-dihydro-1H-imidazole), was docked with the target protein. The binding affinity exhibited was noted as −5.3 kcal/mol. Upon comparison, the molecular docking results of the novel compounds were more favorable than the baseline compound. The molecular interaction showed that the compound formed a hydrogen bond with Asp190. Furthermore, van der Waals interaction was observed with the residues Asp101, Asn120, Met144, Ile122, Leu220, Glu222, Leu226, Arg261, and Phe195, as shown in Figure 4.

### 3.5. ADME Properties and Lipinski’s Rule of Five Profile of Screened Compounds

The drug-likeness and pharmacokinetics profile were evaluated through in silico absorption, distribution, metabolism, and excretion (ADME) analysis. These characteristics are essential for predicting the behavior of selected drugs within the human body [41]. The compounds’ Simplified Molecular Input Line Entry Systems (SMILESs) were obtained from an online SMILES translator tool; afterwards, the ADME analysis was carried out. The top-10 compounds were assessed, and out of these, three compounds met Lipinski’s rule of five criteria with zero violations. The compounds showed a favorable oral bioavailability score. The molecular weight criteria (<500 Da), acceptors (<10), and hydrogen bond donors (<5) were all satisfied. Table S1 offers a detailed summary of the ADME results, which include the pharmacokinetics, drug-likeness, lipophilicity, and Lipinski’s rule of five.

### 3.6. Molecular Dynamics (MD) Simulation

MD simulations were employed in this research to comprehend how atoms in entire macromolecules evolve under physiological environments. This approach can be used to assess protein–ligand complexes’ strength, balance, and pattern of interaction. The MD trajectories after a 100 ns simulation run were analyzed and plots were generated for the root mean square deviation, (RMSD), beta-factor, radius of gyration (RoG), root mean square fluctuation (RMSF), and lastly, solvent accessible surface area (SASA).

**Figure 5:**
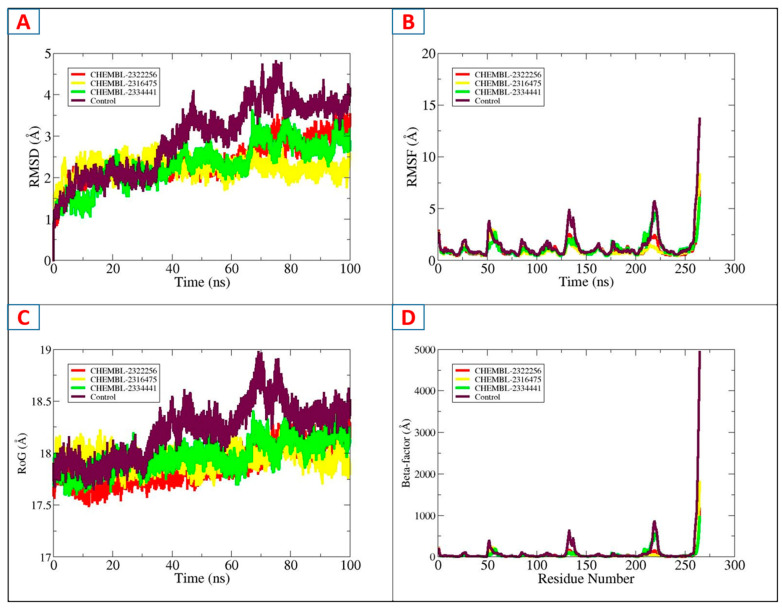
The analysis of the MD trajectories of CHEMBL2322256 (red), CHEMBL2316475 (yellow), CHEMBL2334441 (green), and the control (maroon). (**A**). RMSD, (**B**). RMSF, (**C**). RoG and (**D**). beta factor analysis.

The difference in the atoms’ positions in the protein structure in either of the bound and unbound states, as they underwent MD simulation, is termed the RMSD [42]. The mean RMSD for the DHPS–CHEMBL2322256 complex measured 2.41 Å. Increased structural stability, along with fewer variations, is represented by a lower RMSD score, and vice versa. CHEMBL2316475 and CHEMBL2322256 have been found to show greater structural stability and reduced deviation. Throughout the simulation period, major variations and decreased structural stability were observed in the control complex. In MD simulations, the RMSF is a statistical method that measures the usual fluctuations of the atomic positions from their averages over time [43]. Since it provides details about the shifts and flexibility of a protein’s structure, this parameter is essential for assessing the consistency of proteins in both scenarios. Despite the notable exception of the terminal point (250–270), which showed significant variations in the control complex, as shown in Figure 5B, all of the protein–ligand complexes, even the control, had comparable residue fluctuations. For CHEMBL2322256, CHEMBL2316475, CHEMBL2334441, and the control, the corresponding mean RMSF scores were 1.03 Å, 0.95 Å, 1.32 Å, and 1.43 Å. The radius of gyration (ROG), typically calculated for the protein backbone, can be used to determine the compactness of the protein structure [44]. Evaluating the compactness of the protein structure during simulations in both the unbound and bound positions is eventually necessary to determine the form of ligand that interacts with the protein’s active site and its behavior in a physiological condition. A higher ROG value indicates greater conformational changes, while a lower value indicates less conformational dynamism in the protein’s structural properties. Except for a small spike in the CHEMBL2334441 complex, as illustrated in Figure 5C, which occurred between 65 and 80 ns, the trajectories of the novel complexes exhibited a consistent pattern. For CHEMBL2322256, CHEMBL2316475, CHEMBL2334441, and the control, the corresponding minimum ROG values were 17.49 Å, 17.66 Å, 17.59 Å, and 17.64 Å. To identify the thermal movements of atoms, the beta-factor was analyzed [45]. The control was noted to exhibit higher fluctuations compared to the other complexes. CHEMBL2322256 and CHEMBL2316475 exhibited lower beta-factors, demonstrating strong binding, as shown in Figure 5D.

**Figure 6:**
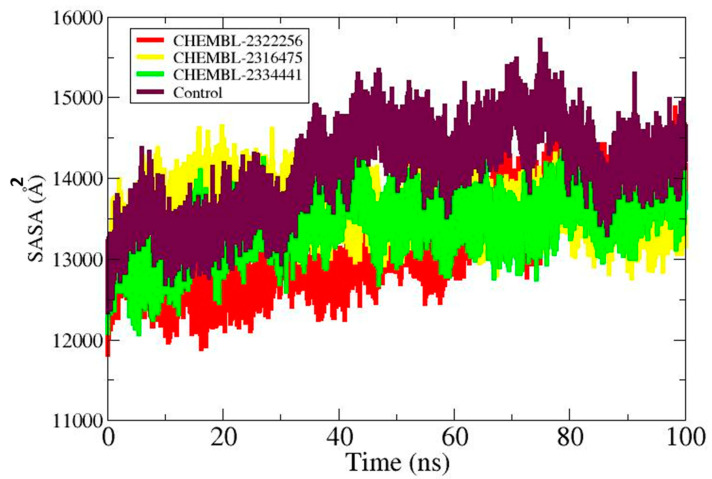
SASA analysis of CHEMBL2322256 (red), CHEMBL2316475 (yellow), CHEMBL2334441 (green), and the control (maroon).

SASA demonstrated that the control–DHPS complex and DHPS–ligand complexes differed in their conformational changes as a result of ligand binding and solvent interactions [46]. Under virtual physiological circumstances, the control–DHPS enzyme exhibited a mean SASA profile of 14,103.1 Å^2^. CHEMBL2322256 (13,237.4 Å^2^), CHEMBL2316475 (13,623.1 Å^2^), and CHEMBL2334441 (13,427.1 Å^2^) exhibited the average SASA profile. The SASA plot for each complex is given in Figure 6.

### 3.7. Hydrogen Bonding Analysis

Post-simulation, an analysis of the hydrogen bonds was carried out between the DHPS and the ligands over the simulation. A key factor in determining the magnitude of binding interactions is hydrogen bonding, especially in the context of protein–ligand interactions [47]. Recognizing the essential role of hydrogen bonding in these processes, we calculated the number of hydrogen bonds throughout each trajectory at different times, which revealed insight into the dynamic nature of these essential interactions. Considering the importance of hydrogen bonding calculations in assessing the stability of the protein–ligand complex, the average total number of hydrogen bonds in each complex was calculated as well. After assessing the data, it became obvious that the DHPS–control complex continuously and more strongly interacted with ASP_172@OD2LIG_266@N1, outperforming the other three complexes with the greatest number of frames (854). The residues of GLU_52@O, LIG_266@H, and LIG_266@N then exhibited the most attractive hydrogen bond interactions with CHEMBL2316475, with an overall bond angle of 159.422 Å, along with a bond distance of 2.8056 Å; 640 frames were measured. Furthermore, CHEMBL2322256 formed hydrogen bonds with the DHPS residues ASP_83@OD2LIG_266@H1 and LIG_266@O4 within the 89 number of frames, showing weak interactions, while CHEMBL2334441 performed moderately, as mentioned in Table 2.

### 3.8. Salt Bridge Formation and Electrostatic Interaction

CHEMBL2316475 demonstrated the most favorable salt bridge formation profile, followed by the CHEMBL2322256 complex. Recurring interactions such as Glu222–Arg217 and Asp218–Arg209 contribute to enhancing the specificity of the control. The substantial amount of electrostatic interactions, particularly varied and repeated ones, in CHEMBL2322256 indicated potential strengthening. CHEMBL2334441 showed several salt bridges. Similarly, to the control complex, its profile displayed different interactions (Glu59–Arg63, Glu52–Lys86). Considering both of the aforementioned in relation to CHEMBL2316475, the salt bridge profile was found to be comparatively stable, as shown in Figures S1 and S2. Table 3 tabulate salt bridges for the complexes.

### 3.9. An Analysis of the Secondary Structure Transitions upon Ligand Binding

**Figure 7:**
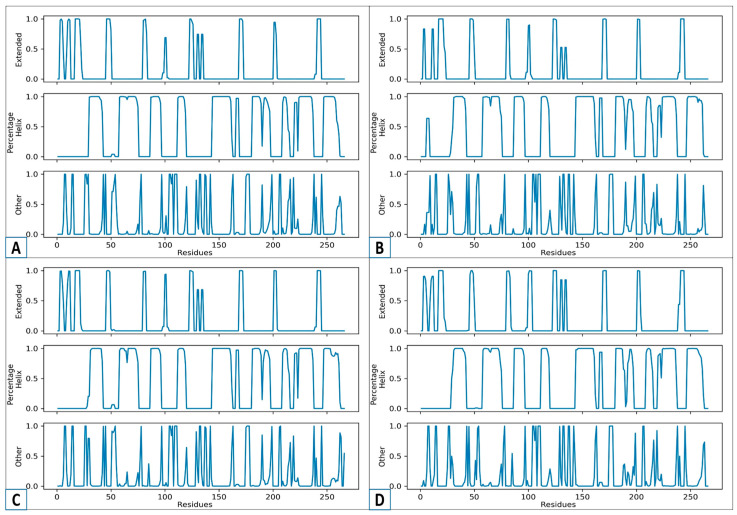
The analysis of the secondary structure changes over the simulation time for the top-three complexes, CHEMBL2322256 (**A**), CHEMBL2316475 (**B**), and CHEMBL2334441 (**C**), and the control (**D**).

Ligand-induced secondary structure analysis illustrates the manner in which the attachment of a small molecule (ligand) modifies the protein’s regular, regional structural characteristics, like alpha-helices and beta-sheets [48]. Both drug development and the study of protein behavior rely on a comprehension of how ligands affect both the form and the function of proteins. MD simulation is the techniques used for detecting these changes [49]. The secondary structure analysis of CHEMBL2322256 (A), CHEMBL2316475 (B), CHEMBL2334441 (C), and the control (D) is displayed in Figure 7. Three sections, including “Extended,” “Percentage Helix,” and “Other,” appear in Figure 7. The “Percentage Helix” component displays the alpha-helix, whilst the “Extended” section is related to the beta-sheets. The structure’s coils, turns, and loops are referred to in the “Other” section. Based on the research, CHEMBL2322256 (A) exhibited a consistent helix and expanded regions. Further, the other area revealed limited fluctuations, showing that the molecule was stable upon ligand binding. In both the helical and extended parts, the profiles of CHEMBL2316475 (B) and CHEMBL2322256 (A) are identical. In contrast to A and B, CHEMBL2334441 (C) is a bit unstable. After 100 residues, the control that served as a standard for the current study displayed a significant decrease in the helix. However, complex A presented the greatest structural stability, followed by B and C. Increased fluctuations, disorganized areas, and less structural flexibility were observed in the control (D).

### 3.10. MMPBS/GBSA Calculations

Docking results can be validated with accuracy, speed, and low computing cost using the binding free energy calculation approach [50]. Therefore, the current study utilized the MMPB/GBSA methodologies to estimate the binding free energy while taking into account the possible advantages of this approach. The MM/GBSA methods were employed for calculating the van der Waals energy of the following complexes: CHEMBL2322256 (−114.20 kcal/mol), CHEMBL2316475 (−98.62 kcal/mol), CHEMBL2334441 (−111.08 kcal/mol), and the control complex (−84.08 kcal/mol). The electrostatic energy was estimated to be −26.38 kcal/mol for the CHEMBL2322256 complex, −22.01 kcal/mol for the CHEMBL2316475 complex, −25.67 kcal/mol for the CHEMBL2334441 complex, and −16.34 kcal/mol for the control complex. Employing the MM/GBSA approach, the average free binding energy for the CHEMBL2322256 complex was found to be −126.49 kcal/mol, −105.63 kcal/mol (CHEMBL2316475), −65.57 kcal/mol (CHEMBL2334441), and −85.04 kcal/mol (control). Further, the MMPB/SA method generated net binding free energies of −124.49 kcal/mol, −103.75 kcal/mol, −65.28 kcal/mol, and −83.62 kcal/mol for CHEMBL2322256, CHEMBL2316475, CHEMBL2334441, along with the control, correspondingly. The results showed that according both the MMPB/GBSA techniques, CHEMBL2322256 is a highly stable complex with the smallest negative binding energy. In contrast to the novel complexes, the control as the baseline complex showed less favorable findings, suggesting weaker binding. The binding free energy findings derived from the MMPB/GBSA method are presented in Table 4 This lends further support to DHPS’s potential as an effective treatment for drug resistance. The binding free energy figures derived from the MMPB/GBSA techniques are summarized in Table 4.

### 3.11. Entropy Energy Estimation

The calculation attempts to determine a system’s level of disorder and randomness [51]. Knowing how this randomness varies and changes in response to protein folding and ligand binding is also important. Data regarding the three complexes and control are shown in Table 5. The table includes the vibrational, translational, and rotational entropy values of the protein–ligand complexes. With fewer problems and less randomness in a chemical system, CHEMBL2322256 exhibited the most favorable value of 8.63 kcal/mol, depending on the findings. With less disorder, CHEMBL2316475 (15.03 kcal/mol) was the second most stable complex identified. The highly stable complex, CHEMBL2322256, confirmed the MMPB/GBSA results and encouraged its selection as a potential therapeutic candidate. The control was considerably less stable than the previously mentioned complexes, with an entropy energy estimate of 16.33 kcal/mol.

## 4. Discussion

The Gram-negative, non-fermenting bacterium *S. maltophilia* has become an opportunistic nosocomial pathogen [7]. Treatment of bacterial infections is extremely challenging due to their inherent antibiotic resistance [15]. The lack of defined breakpoints for the few antibiotics that have in vitro activity against this microbe, the inequalities in the spread of antibiotic resistance as well as the virulence factors among strains, and the limitations of current antimicrobial susceptibility tests all contribute to clinical management [52]. The methods of in vitro studies and in vivo investigations are laborious and costly [53]. However, in silico methods can effectively and rapidly detect potential inhibitors, making up for the shortcomings of experimental methods [54]. For making accurate predictions, the computational techniques use a range of algorithms and sequence, as well as structure-based prediction techniques [55].

The current study targeted DHPS, the main enzyme in the folate biosynthesis pathways in bacteria, which catalyzes the condensation of para-amino benzoic acid with dihydropterin pyrophosphate (DHPP) to form dihydropteroate, a precursor of tetrahydrofolate, which is vital for bacterial protein, RNA and DNA synthesis [56]. *S. maltophilia* is a multidrug-resistant bacteria, increasingly related to infections in immunocompromised patients. The infections include bloodstream infections, cystic fibrosis and pneumonia [7]. Sulfonamide antibiotics, such as sulfamethoxazole, cannot bind when the DHPS enzyme is mutated, rendering the treatment ineffective [57]. Hence, novel inhibitors are needed to combat drug resistance. The current research utilized an integrated computational approach to target DHPS, the main enzyme involved in folate biosynthesis pathway. A comprehensive computer-aided study was executed in the present investigation by screening the CHEMBL library, which contains 2496 compounds, against DHPS. Based on their potential binding affinity score, the top-10 compounds were chosen after the docking studies were analyzed. The binding score of a control, however, was −5.3 kcal/mol. Afterwards, the ADME analysis was performed, selecting the top-three compounds as CHEMBL2322256, CHEMBL2316475, and CHEMBL2334441, revealing remarkable ADMET characteristics. The compound CHEMBL2322256, with the chemical name 2-(4,11-dimethyl-2-oxo-6,7,8,9-tetrahydro-2H-benzofuro[3,2-g]chromen-3-yl)-N-(3-hydroxyphenyl)acetamide, features coumarins and derivatives with the subclass of benzofurochromenones. The class structure has been recognized for many antibacterial activities. The compound CHEMBL2316475, with the chemical name 8-((2, 4-difluorophenyl)amino)-N-(2-methoxyethyl)-5-oxo-10, 11-dihydro-5H-dibenzo[a,d][7]annulene-3-carboxamide, having antibacterial activity, features polycyclic aromatic hydrocarbons with the subclass of dibenzo annulenes. The compound is well known for its antibacterial functions. CHEMBL2334441, having chemical name 8-methoxy-5-methyl-10-(2-phenylhydrazinyl)-1,2,3,5,6,7,8,9-octahydroindeno[1,2-b]indole, features fused indole systems with the subclass of indeno[1,2-b]indoles [58]. The compounds were deemed drug-like because they satisfied with the Lipinski criteria. To investigate the stability of DHPS and the compounds in a dynamic environment, an MD simulation of 100 ns was performed subsequently. This demonstrates conformational changes at the binding position, changes that are not visible during the static docking procedure, as well as the continuous stability of the binding contacts [59]. The results of the hydrogen bond and salt bridge investigations provide essential details about the inhibitor’s likelihood of therapeutic use. After analyzing the hydrogen bonding of the three complexes and control, it was apparent that the DHPS–control complex consistently and more firmly interacted with ASP_172@OD2LIG_266@N1, outperforming the other three complexes with the maximum number of frames (854). Consequently, the residues that CHEMBL2316475 exhibited the most efficient hydrogen bond interactions with were GLU_52@O, LIG_266@H, and LIG_266@N. With an average bond angle of 159.422 Å, along with bond distance of 2.8056 Å, 640 frames were recorded. The newly identified compounds formed stronger complexes with DHPS versus the control, as determined by the salt bridge formation and secondary structure studies. Computational drug design relies heavily on salt bridge analysis for multiple reasons. Salt bridges occur between oppositely charged residues of amino acids to stabilize molecular interactions and protein structures [60]. Researchers may utilize computational analysis of salt bridges to identify crucial interactions needed for drug binding, which can assist them in better understanding both the stability and the mobility of protein–ligand complexes. Better structural compatibility is shown with minimal modification of the protein’s secondary structure, while stronger electrostatic stabilization is suggested by more salt bridge contacts. All of these results support the new ligands’ potential as powerful DHPS inhibitors against *S. maltophilia* by verifying that they increase structural stability. The docking studies of the DHPS–compound complexes were validated by MMPB/GBSA approaches. The MMPB/GBSA showed the highest score of −126.49/−124.49 kcal/mol for CHEMBL2322256. However, the net energy calculated for the control (−85.04/−83.6 kcal/mol) was less than the novel identified inhibitors. The entropy energy verified that CHEMBL2322256 is the most stable DHPS complex, with the entropy energy estimate of 8.63 kcal/mol.

One of the studies that aligns with the current research was conducted by [61], focusing on identifying DHPS inhibitors against *Acinetobacter baumannii.* The study adopted an in silico approach. Using in-house libraries of natural compounds from medicinally significant plants and *Agaricus* spp. fungi, the active site of the DHPS enzyme was computationally screened for antimicrobial agents. Following multiple screenings using Lipinski’s dependent drug-like criteria, pharmacokinetic parameters, toxicity parameters, and structural parameters—which included the calculated free energy of binding, ligand efficiency, and interaction analysis—two ligands (CID_291096 and MSID_000725) were identified as suitable candidate inhibitors. In the bacterial cell’s folic acid production pathway, the DHPS enzyme facilitates the condensation reaction between para-amino benzoic acid and hydroxymethyl-7,8-dihydropterin pyrophosphate. In silico investigations, including MD simulations and MM/PBSA-based binding free energy studies, are used to validate the DHPS enzyme and ligand complexes. One of the related studies was performed by [62] with the aim of identifying inhibitors against DHPS of *Helicobacter pylori*. The study utilized different computational approaches, such as molecular docking, MD simulation, ADME properties, and binding free energy estimation. Upon comparing the findings of the present study with those of previous reported studies, it was evident that the previous studies were based on sulfonamide-based scaffolds, while the present study did not contain a sulfonamide group. This suggests that the identified compounds are novel and could combat drug resistance related to sulfonamide group rings.

Substantial testing should be carried out to evaluate the safety and effectiveness of the lead chemicals found in computational research. Even though in silico evaluation has a lot of potential, this study has limitations. The simulation parameters and the overall state of the protein’s structure have the most effect on the computational accuracy. Docking studies face the risk of ignoring the effects of solvents and the flexibility of proteins, which could result in false-positive results. The inhibitors’ efficacy as lead molecules for therapeutic development needs to be verified and confirmed through experimental research.

## 5. Conclusions

The need to discover new antimicrobial agents has become crucial due to the growing trend of antibiotic resistance. Many health agencies have classified *S. maltophilia* as a severe health danger; thus, we focused on it in this study. In this research, the DHPS enzyme—a crucial biocatalyst in bacterial survival mechanisms—was virtually screened and targeted against a CHEMBL library. Following that, the compounds underwent interaction analysis and molecular docking study using AutoDock to narrow down their shortlist. The pharmacokinetics assessment shortlisted three ligands: CHEMBL2322256, CHEMBL2316475, and CHEMBL2334441. These ligands demonstrated reliable interaction via bond formation when they interacted with the DHPS enzyme. The control was used a benchmark in the current research. The MD simulation variables, including the Rg, SASA, RMSD, and RMSF, assessed both ligands as possible therapeutic options. Therefore, these inhibitors may be recognized as possible therapeutic possibilities, with CHEMBL2322256 and CHEMBL2316475 binding more impulsively. Though the results are promising and may provide excellent leads for additional structure and biological activity optimization, experimental validation is needed to validate the findings. These options, however, are generally not capable of accurately simulating real physiological conditions. Therefore, for validating the in silico-driven concept, more experimental research as well as preclinical and clinical tests are required. Furthermore, before being taken into consideration for future development as a viable drug molecule, natural compounds that were shown to be potential drug candidates in preclinical investigations should go through a rigorous validation process.

## Figures and Tables

**Table 1:** The top-10 shortlisted compounds from docking studies against DHPS. The compounds are shown, along with their binding affinity score, chemical structure, and name. Furthermore, it includes a control (imidazole), utilized as a baseline compound.

Rank	Compound ID	Binding Affinity	Chemical Name and Structure
1.	CHEMBL2322256	−8.3 kcal/mol	2-(4,11-dimethyl-2-oxo-6,7,8,9-tetrahydro-2H-benzofuro[3,2-g]chromen-3-yl)-N-(3-hydroxyphenyl)acetamide 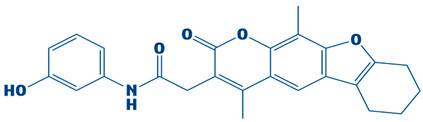
2.	CHEMBL2316475	−7.8 kcal/mol	8-((2,4-difluorophenyl)amino)-N-(2-methoxyethyl)-5-oxo-10,11-dihydro-5H-dibenzo[a,d][7]annulene-3-carboxamide 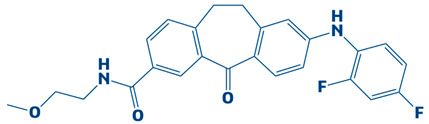
3.	CHEMBL2334441	−7.6 kcal/mol	8-methoxy-5-methyl-10-(2-phenylhydrazinyl)-1,2,3,5,6,7,8,9-octahydroindeno[1,2-b]indole 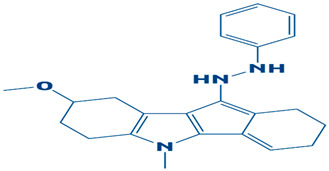
4.	CHEMBL2334176	−7.5 kcal/mol	5-(1-(2,3-dimethylbenzoyl)piperidin-3-yl)-3-phenyl-4,5-dihydro-1,2,4-oxadiazol-2-ium 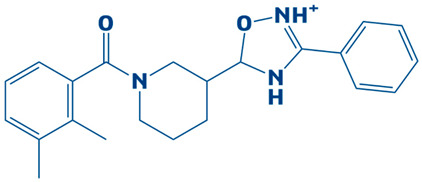
5.	CHEMBL2334557	−7.5 kcal/mol	4-amino-5-((4-(tert-butyl)phenyl)ethynyl)-1-(3,4,5-trihydroxy-6-(hydroxymethyl)tetrahydro-2H-pyran-2-yl)pyrimidin-1,3-diium-2-olate 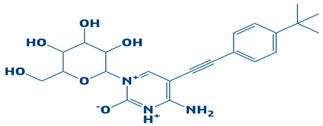
6.	CHEMBL15935	−7.4 kcal/mol	4-((3-(4-methylpiperazin-1-yl)propyl)carbamoyl)benzyl 2-oxo-6-propyl-4-(m-tolyl)-1,2,3,4-tetrahydropyrimidine-5-carboxylate 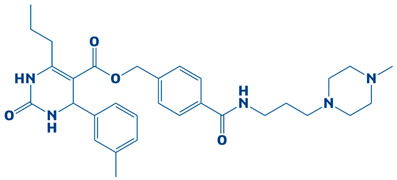
7.	CHEMBL2260150	−7.3 kcal/mol	3-((3-(4,4-diphenylpiperidin-1-yl)propyl)carbamoyl)-5-(methoxycarbonyl)-4-(4-methoxyphenyl)-2,6-dimethylpyridin-1-ium 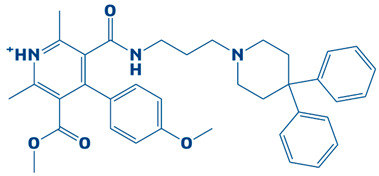
8.	CHEMBL2322867	−7.3 kcal/mol	2-(5-(hydroxymethyl)-4-methyl-3-(trifluoromethyl)-2,3-dihydro-1H-pyrazol-1-yl)-1-(4-(3-methoxy-4-methylphenyl)piperazin-1-yl)ethanone 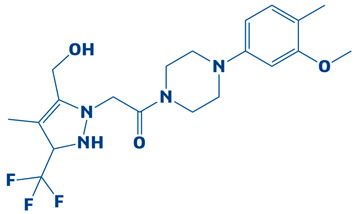
9.	CHEMBL2334162	−7.3 kcal/mol	5-(1-(1-methyl-1H-indole-4-carbonyl)piperidin-3-yl)-3-phenyl-4,5-dihydro-1,2,4-oxadiazol-2-ium 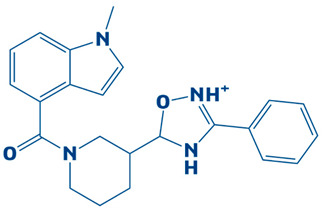
10.	CHEMBL2229522	−7.2 kcal/mol	(E)-1-(furan-3(2H)-ylidene)-4,7a-dimethyl-3-oxo-1,3,5,6,7,7a-hexahydroisobenzofuran-5-yl 4-cyanobenzoate 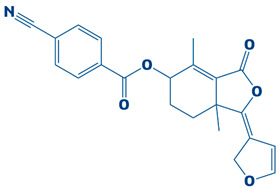
11.	Imidazole (Control)		2,3-dihydro-1H-imidazole 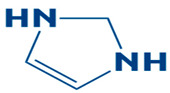

**Table 2:** The hydrogen bond interaction between the DHPS residues and the top hits. It consists of the main hydrogen bonds formed between the residues, along with the number of frames, average bond distance and angle. AvgDist (average distance) and AvgAng (average angle).

Control						
#Acceptor	DonorH	Donor	Frames	Frac	AvgDist	AvgAng
ASP_172@OD2	LIG_266@HN	LIG_266@N1	854	0.854	2.8301	162.8601
ASP_83@OD2	LIG_266@H	LIG_266@N	274	0.274	2.8473	160.1394
ASP_83@OD1	LIG_266@H	LIG_266@N	114	0.114	2.8507	158.6697
LIG_266@N1	ASN_102@HD22	ASN_102@ND2	52	0.052	2.9458	155.5515
ASN_102@O	LIG_266@H	LIG_266@N	39	0.039	2.9068	153.4423
LIG_266@N1	ARG_243@HH11	ARG_243@NH1	1	0.001	2.915	135.4934
**CHEMBL2322256**						
**#Acceptor**	**DonorH**	**Donor**	**Frames**	**Frac**	**AvgDist**	**AvgAng**
ASP_83@OD2	LIG_266@H1	LIG_266@O4	89	0.089	2.805	156.8071
LIG_266@O3	HIE_245@HE2	HIE_245@NE2	21	0.021	2.906	146.6492
GLY_204@O	LIG_266@H1	LIG_266@O4	15	0.015	2.8061	159.5714
ARG_243@NH1	LIG_266@H1	LIG_266@O4	12	0.012	2.9592	153.7587
LIG_266@O3	ASN_23@HD21	ASN_23@ND2	7	0.007	2.9273	153.1301
ASP_172@OD1	LIG_266@H1	LIG_266@O4	1	0.001	2.8756	162.7102
ARG_243@NH2	LIG_266@H1	LIG_266@O4	1	0.001	2.9119	136.3553
**CHEMBL2316475**						
**#Acceptor**	**DonorH**	**Donor**	**Frames**	**Frac**	**AvgDist**	**AvgAng**
GLU_52@O	LIG_266@H	LIG_266@N	640	0.64	2.8056	159.422
GLY_176@O	LIG_266@H1	LIG_266@N1	46	0.046	2.8677	148.7695
GLU_52@OE1	LIG_266@H	LIG_266@N	29	0.029	2.8047	153.4161
LIG_266@O2	GLY_178@H	GLY_178@N	5	0.005	2.8986	156.9123
LIG_266@O1	LYS_208@HZ2	LYS_208@NZ	1	0.001	2.793	150.6578
LIG_266@O1	LYS_208@HZ3	LYS_208@NZ	1	0.001	2.9694	138.0812
LIG_266@F1	HIE_134@HE2	HIE_134@NE2	1	0.001	2.9953	137.6677
**CHEMBL2334441**						
**#Acceptor**	**DonorH**	**Donor**	**Frames**	**Frac**	**AvgDist**	**AvgAng**
GLU_52@OE1	LIG_266@HN	LIG_266@N1	156	0.1563	2.8373	156.3745
GLU_52@OE2	LIG_266@HN	LIG_266@N1	155	0.1553	2.8607	154.4868
GLU_52@O	LIG_266@HN	LIG_266@N1	8	0.008	2.8815	148.5877
GLY_133@O	LIG_266@H	LIG_266@N2	5	0.005	2.8763	151.1339

**Table 3:** The salt bridge formation of the top-three DHPS–complexes, CHEMBL2322256, CHEMBL2316475, and CHEMBL2334441, and the control.

S.No	DHPS–Ligand Complex	Electrostatic Interactions
1.	CHEMBL2322256	Asp252-Lys255, Asp218-Arg223, Asp252-Lys255, Glu60-Arg63, Glu261-Arg240, Glu60-Arg63, Glu160-Lys166, Asp218-Arg209, Asp218-Arg209, Asp218-Arg209, Glu222-Arg217, Glu60-Arg63, Asp143-Arg190, Glu222-Arg217, Asp143-Arg190, Glu2130arg223, Glu60-Arg63, Glu60-Arg64, Asp246-Arg207, Glu222-Arg217, Glu192-Arg237, Glu196-Arg193,Asp14-Arg12, Glu60-Arg64, Glu213-Arg209, Glu213-Arg209, Glu213-Arg209, Asp5-Lys167, Asp218-Arg223, Glu42-Lys38.
2.	CHEMBL2316475	Asp252-Lys255, Asp45-Arg17, Asp83-Arg243, Glu192-Arg193, Glu61-Lys86, Glu60-Arg63, Glu59-Arg63, Glu192-Arg193, Glu61-Lys86, Asp48-Arg243, Glu88-Arg91, Asp143-Arg190, Asp218-Arg209, Asp143-Arg190, Glu88-Arg91, Glu52-Lys86, Glu60-Arg64, Glu59-Arg63, Asp246-Arg207, Glu60-Arg64, Asp45-Arg15, Glu192-Arg190, Glu196-Arg193, Glu192-Arg190, Glu196-Arg193, Glu192-Arg237, Glu192-Arg237, Glu192-Arg190,Glu192-Arg190, Asp14-Arg17, Glu213-Arg209, Asp14-Arg17, Asp45-Arg15, Asp14-Arg12, Glu42-Lys38.
3.	CHEMBL2334441	Asp252-Lys255, Asp14-Arg17, Asp252-Lys255, Glu60-Arg63, Glu42-Lys38, Asp83-Arg243, Glu160-Lys166, Asp218-Arg209, Asp14-Arg15, Glu88-Arg91, Glu160-Lys166, Asp143-Arg190, Asp246-Arg207, Asp48-Arg243, Glu41-Lys38, Asp14-Arg15, Asp246-Arg207, Asp246-Arg207, Asp138-Arg217, Asp138-Arg209, Glu192-Arg237, Glu213-Arg209, Glu213-Arg209, Asp14-Arg17, Glu213-Arg219, Glu213-Arg209, Asp5-Lys167, Asp218-Arg223, Asp14-Arg17.
4.	Control	Asp252-Lys255, Asp45-Arg17, Glu261-Arg240, Glu131-Arg108, Glu61-Lys86, Glu60-Arg63, Glu41-Lys38, Asp218-Arg209, Asp218-Arg209, Asp218-Arg209, Glu222-Arg217, Asp143-Arg190, Glu222-Arg217, Asp218-Arg209, Asp48-Arg243, Glu192-Arg237,3 Glu60-Arg64, Glu88-Arg91, Asp48-Arg243, Glu88-Arg91, Glu196-Arg193, Asp246-Arg207, Asp45-Arg15, Asp14-Arg12, Glu60-Arg64, Glu60-Arg64, Glu213-Arg209, Asp14-Arg12, Asp218-Arg223, Glu30-Arg75.

**Table 4:** The binding free energy for the top-three hits and control, via the MMPB/GBSA methods for CHEMBL2322256, CHEMBL2316475, CHEMBL2334441, and the control.

Energy Parameter	CHEMBL2322256	CHEMBL2316475	CHEMBL2334441	Control
MM/GBSA				
Energy van der Waals	−114.20	−98.62	−111.08	−84.08
Energy electrostatic	−26.38	−22.01	−25.67	−16.34
Total gas phase energy	−140.58	−120.63	−85.41	−100.42
Total salvation energy	14.09	15.00	19.84	15.38
Net energy	−126.49	−105.63	−65.57	−85.04
**MMPB/SA**				
Energy van der Waals	−114.20	−98.62	−111.08	−84.08
Energy electrostatics	−26.38	−22.01	−25.67	−16.34
Total gas phase energy	−140.58	−120.63	−85.41	−100.42
Total energy salvation	16.09	16.88	20.13	16.82
Net energy	−124.49	−103.75	−65.28	−83.6

**Table 5:** The entropy energy estimation and randomness via different algorithms, such as translational, vibrational, and rotational, for CHEMBL2322256, CHEMBL2316475, CHEMBL2334441, and the control.

Complex	Translational	Vibrational	Rotational	DELTA S Total
CHEMBL2322256	16.35	1023.01	18.64	8.63
CHEMBL2316475	15.14	984.05	19.37	15.03
CHEMBL2334441	18.06	1102.36	21.01	18.01
Control	20.59	975.05	19.80	16.33

## Data Availability

The original contributions presented in the study are included in the article; further inquiries can be directed to the corresponding author.

## Supplementary Materials

The following supporting information can be downloaded at https://www.mdpi.com/article/10.3390/biology14081030/s1, Figure S1: The salt bridges formation profile of CHEMBL2322256 (A), CHEMBL2316475 (B).; Figure S2: The salt bridges interaction of CHEMBL2334441 (C) and Control (D). Table S1: The top three compounds’ pharmacokinetic features are CHEMBL2322256, CHEMBL2316475, CHEMBL2334441, and Control.
