# Supplementary material for: Integrating Multi-Domain Approach for Identification of Neo Anti-DHPS Inhibitors Against Pathogenic Stenotrophomonas maltophilia
Source: Biology (Basel). 2025 Aug 11;14(8):1030. doi: 10.3390/biology14081030 (PMC12383866; doi:10.3390/biology14081030)
Supplement: Supplementary file 1 [file biology-14-01030-s001.zip › biology-3797022-supplementary.pdf]

**Supplementary File**

**S-Table 1.** The top three compounds' pharmacokinetic features are CHEMBL2322256, CHEMBL2316475, CHEMBL2334441, and Control.

| **PHYSIOCHEMICAL PROPERTIES** | | | | | | | | | |
|---|---|---|---|---|---|---|---|---|---|
| **Compounds** | **Formula** | **Molecular weight** | **Num. heavy atoms** | **Num. aroma. heavy atoms** | **Fraction Csp3** | **Num. H-bond acceptors** | **Num. -bond donors** | **Molar Refractivity** | **TPSA** |
| CHEMBL2322256 | C25H23NO5 | 417.45g /mol | 31 | 19 | 0.28 | 5 | 2 | 120.44 | 92.68 Å |
| CHEMBL2316475 | C25H22F2N | 436.45 g/molg/mol | 32 2 g/molg/mol | 18 | 0.20 | 5 | 2 | 117.29 | 67.43 Å$^{22}$ |
| CHEMBL2334441 | C23H27 N3O | 361.48 g/mol | 27 | 11 | 0.39 | 4 | 1 | 111.02 | 38.22 Å$^{2}$ |
| **Control** | C3H6N2 | 70.09 g/mol | 5 | 0 | 0.33 | 0 | 0 | 27.38 | 24.06 Å$^{2}$ |

| **LIPOPHILICITY** | | | | | | |
|---|---|---|---|---|---|---|
| **Compounds** | **Log $P_{o/w}$ (iLOGP)** | **Log $P_{o/w}$ (XLOGP3)** | **Log $P_{o/w}$ (WLOGP)** | **Log $P_{o/w}$ (MLOGP)** | **Log $P_{o/w}$ (SILICOS-IT)** | **Consensus Log $P_{o/w}$** |
| CHEMBL2322256 | 3.26 | 4.25 | 4.73 | 2.97 | 5.81 | 4.20 |
| CHEMBL2316475 | 3.51 | 4.52 | 5.25 | 3.55 | 5.67 | 4.50 |

| | | | | | | |
|---|---|---|---|---|---|---|
| CHEMBL2334441 | 3.67 | 4.10 | 4.25 | 3.35 | 3.52 | 3.78 |
| **Control** | 0.67 | 2.34 | -1.15 | -0.07 | 0.38 | -0.11 |

**PHARMACOKINETICS**

| **Compounds** | **GI absorption** | **BBB permeant** | **P-gp substrate** | **CYP1A2 inhibitor** | **CYP2C19 inhibitor** | **CYP2C9 inhibitor** | **CYP2D6 inhibitor** | **CYP3A4 inhibitor** | **Log $K_p$ (skin permeation)** |
|---|---|---|---|---|---|---|---|---|---|
| CHEMBL2322256 | High | NO | Yes | Yes | Yes | Yes | No | No | -5.83 cm/s |
| CHEMBL2316475 | High | No | Yes | Yes | Yes | No | Yes | Yes | -5.75cm/s |
| CHEMBL2334441 | High | Yes | Yes | Yes | Yes | Yes | Yes | Yes | -5.59 cm/s |
| **Control** | Low | No | No | No | No | No | No | No | -6.49 cm/s |

**DRUG LIKENESS**

| **Compounds** | **Lipinski** | **Ghose** | **Veber** | **Egan** | **Muegge** | **Bioavailability Score** |
|---|---|---|---|---|---|---|
| CHEMBL2322256 | Yes; 0 Violation | Yes | Yes | Yes | Yes | 0.55 |
| CHEMBL2316475 | Yes; 0 violation | Yes | Yes | Yes | Yes | 0.55 |
| CHEMBL2334441 | Yes; 0 violation | Yes | Yes | Yes | Yes | 0.55 |

| | | | | | | |
|---|---|---|---|---|---|---|
| **Control** | No; 0 violations: | No; 4 violations: MW<160, WLOGP<-0.4, MR<40, #atoms<20 | Yes | Yes | No; 2 violations: MW<200, #C<5 | 0.55 |

**MEDICINAL CHEMISTRY**

| **Compounds** | **PAINS** | **Brenk** | **Lead likeness** | **Synthetic accessibility** |
|---|---|---|---|---|
| CHEMBL2322256 | 0 alert | 1 alert | No, 2 violations | 3.89 |
| CHEMBL2316475 | 0 alerts | 0 alert | No; 2 violation | 3.43 |
| CHEMBL2334441 | 0 alert | 0 alert: | No; 2 violations | 4.74 |
| **Control** | 0 alert | 0 alert | No; 1 violation: | 2.68 |

**Salt Bridges Formation:**

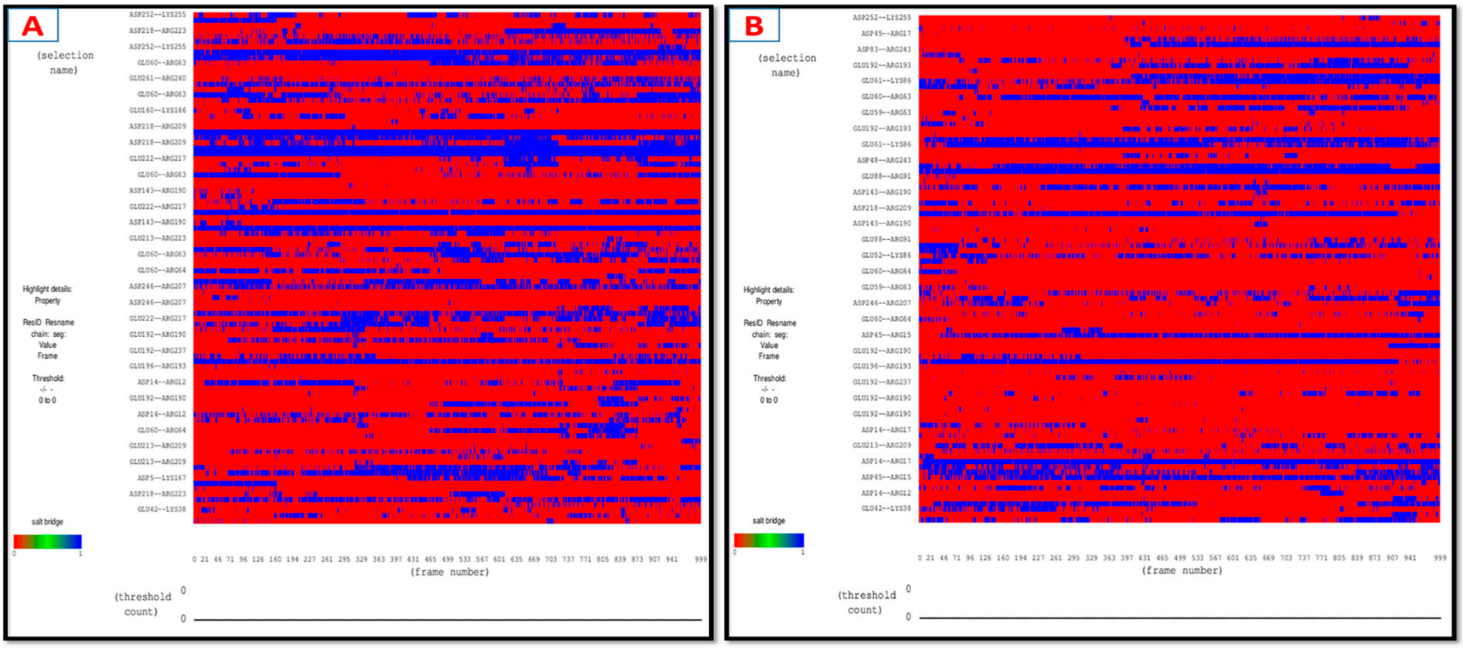


**Figure S-1:** The salt bridges formation profile of CHEMBL2322256 (A), CHEMBL2316475 (B).

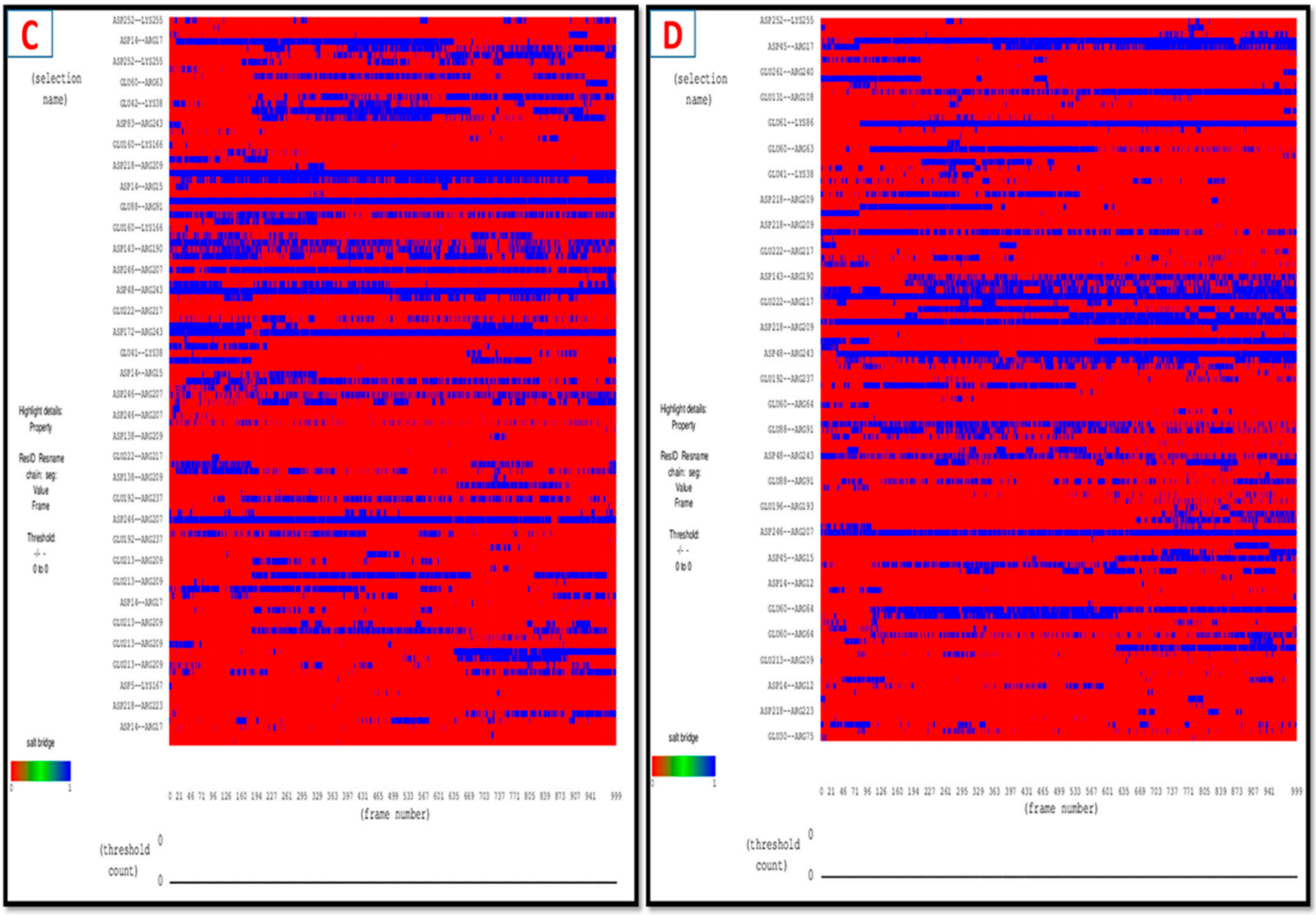


**Figure S-2:** The salt bridges interaction of CHEMBL2334441 (C) and Control (D).
